# Polyphenol-rich oolong tea alleviates obesity and modulates gut microbiota in high-fat diet-fed mice

**DOI:** 10.3389/fnut.2022.937279

**Published:** 2022-07-28

**Authors:** Ang Li, Jin Wang, Ruixin Kou, Mengshan Chen, Bowei Zhang, Yan Zhang, Jingmin Liu, Xiaolong Xing, Bo Peng, Shuo Wang

**Affiliations:** Tianjin Key Laboratory of Food Science and Health, School of Medicine, Nankai University, Tianjin, China

**Keywords:** oolong tea, polyphenol, high-fat diet, obesity, gut microbiota

## Abstract

Obesity is a major public health issue worldwide. Oolong tea (OT), which is partially fermented from *Camellia sinensis* leaves, has proven health benefits and potential preventive applications in multiple studies. However, research on the role of OT in obesity prevention and potential mechanisms is still limited. The purpose of this study was to investigate the modulatory effects of OT intervention on high-fat diet (HFD)-induced obesity and gut microbiota dysbiosis using an obese mouse model. Our results showed that 8-week OT supplementation with 93.94% polyphenols significantly decreased body weight gain, adipose tissue mass, and serum levels of triglyceride (2.60 mmol/L), cholesterol (5.49 mmol/L), and low-density lipoprotein cholesterol (0.61 mmol/L) in HFD-fed mice. Meanwhile, OT intervention was observed to improve fat accumulation, hepatic damage, glucose intolerance, and endotoxemia and alleviate inflammation by decreasing the levels of pro-inflammatory factors. OT also upregulated the expression of genes including *Srebf1*, *Ppara*, *Lxra*, *Pgc1a*, and *Hsl* and downregulated the expression of genes including *Leptin*, *Il-6*, and *Il-1b*. In addition, the gut dysbiosis characterized by decreased flora diversity and increased Firmicutes/Bacteroidetes ratio in obese mice was recovered by OT intervention. Certain differentially abundant microbes caused by HFD feeding, including *Enterococcus*, *Intestinimonas*, *Blautia*, and *Bilophila*, were also improved by OT treatment. This study demonstrated that OT, as a novel resource of dietary polyphenols, exhibited a protective effect on HFD-induced obesity and gut microbiota disorder.

## Graphical Abstract

Regulatory effect and potential mechanisms of oolong tea on obesity in a mouse model. 
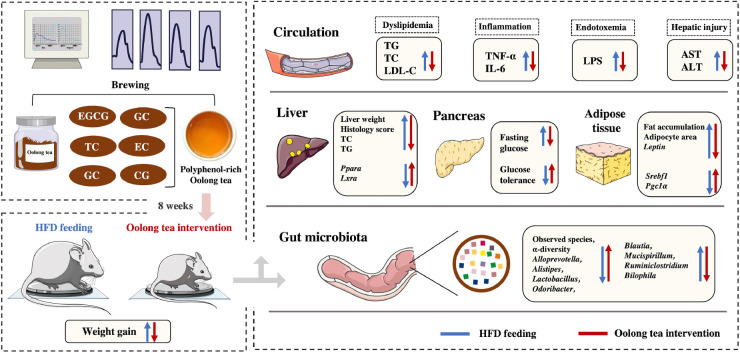

## Introduction

As a growing number of countries adopt high-fat diet (HFD) in modern times, obesity characterized by lipid accumulation, dyslipidemia, metabolic endotoxemia, systematic inflammation, and intestinal dysfunction, has become the fifth greatest global threat to human health in the world (1). The global prevalence rate is 11% in male and 15% in female individuals (2). Increasing evidence also suggests obesity contributes to the development of steatohepatitis, cardiovascular disease (CVD), and certain cancers (3–5). At present, commonly used measures such as physical improvement, calorie control, and pharmacological intervention in obese patients are challenged by polypharmacy and reduced compliance (6). Novel strategies to prevent obesity are thus critically needed. Intriguingly, mounting evidence has demonstrated that gut microbiota is associated with immunity, metabolism, and neurobehavior (7). Several fecal microbiota transplant (FMT) studies have suggested the causal role of gut microbiota in transferring obesity-related symptoms such as weight gain, lipid accumulation, and insulin resistance, proving that gut flora dysbiosis is an important cause of obesity (8). Therefore, targeting the gut microbiota is a promising therapeutic strategy to prevent and treat obesity.

Diets have been considered as one of the most effective methods to regulate gut flora, especially the diet rich in polyphenols has been proved to attenuate obesity (9). Tea, as a polyphenol-rich functional beverage, is brewed from *Camellia sinensis* leaves and consumed by two-thirds of the world’s population due to its high-quality flavor and potential health benefits such as antioxidant, anticancer, antibacterial, and hepatoprotective effects (10). Compared to green tea (unfermented) and black tea (completely fermented), oolong tea (OT) (10–70% partially fermented) has become the focus of consumer attention in eastern countries because of its special flavors such as enhanced semi-oxidized elegant fruity and floral aromas (11). Furthermore, OT also has a variety of health gains, especially the regulation of metabolic disorders. Miyata et al. found the flavins from OT markedly decreased serum triglyceride and elevated fecal fat excretion in animal models (12), and two other clinical trials also demonstrated that OT significantly inhibited weight gain and lipid accumulation and promoted antioxidant and hepatoprotective activities, and the effect was approximately doubled compared with that effect of green tea (13, 14). However, among different types of tea, the health benefits and underlying mechanisms of OT on HFD-induced obesity in mice have been not extensively studied. In addition, considering the bioactive components of OT cannot be absorbed in the small intestine because of their limited bioavailability, we thus speculate that most beneficial ingredients reach the large intestine where they can be metabolized by gut microbes and modulate host metabolisms (1).

In the present study, the main bioactive ingredients such as total polyphenols [catechin (C) and gallic acid (GA)], theanine, and tea protein were characterized in OT. The HFD-fed mice model was established to explore the preventive effects of OT intervention the obesity profile including lipid accumulation, dyslipidemia, glucose intolerance, low-grade inflammation, and intestinal dysfunction. 16S ribosomal RNA (rRNA) sequencing was also applied to investigate the impact of OT on the gut microbiota composition. Overall, our findings will not only provide new insights into potential preventive mechanisms of OT on obesity but also identify the association between the beneficial effect of OT and gut microbiota, which can be used to develop a functional tea beverage for ameliorating obesity and obesity-related diseases.

## Material and methods

### Preparation and characterization of oolong tea

Oolong tea (cultivar of ‘Shuixian’) was obtained from Wuyistar Tea Industrial Co., Ltd. (Fujian, China). Briefly, fresh tea leaves were plucked from *C. sinensis* in October 2020, and the manufacture of OT can be summarized as solar withering, indoor withering, shaking, de-enzyming, rolling, and drying according to the traditional processing of fresh tea leaves into final OT products (11). A measure of 2.5 g OT was brewed for 40 min using 100 mL of 90°C water, and then the filtrate was obtained after fourfold dilution with pure water and cooled to room temperature before testing and feeding to the animals. Tea protein was monitored by using Lowry assay, and quantitative determination of total phenolics was performed using the Folin–Ciocalteu method, as described previously (15, 16). The concentration of theanine, GA, and catechins including epicatechin (EC), gallocatechin gallate (GCG), gallocatechin (GC), epigallocatechin-3-gallate (EGCG), catechin gallate (CG), epicatechin-3-gallate (ECG), and catechin (C) was determined by using an high-performance liquid chromatography (HPLC) system with an Agilent ZORBAX SB-AQ column (250 × 4.6 mm, 5 μm) at 35°C according to Li et al. with minor modifications (17). The mobile phase consisted of 0.2% (v/v) acetic acid (A) and acetonitrile (B) in water with a linear gradient elution, 0–15 min: 3–10% B; 15–20 min: 10–20% B; 15–20 min: 10–20% B; 20–25 min: 20–25% B; 25–30 min: 25–30% B; 30–35 min: 30–35% B; 35–40 min: 35–3% B; 40–45 min: 3% B. Elution was performed with a flow rate of 1 mL/min, and the sample was spectrophotometrically assayed at 270 nm in triplicates.

### Animal experiments

In total, 36 male C57BL/6 mice obtained from Vital River Laboratories (Peking, China) were housed in nine cages under constant temperature (20 ± 1°C) and humidity (55 ± 5%) and able to freely access food and water. After 1-week acclimatization, the mice were randomized into three groups (*n* = 12): normal-diet (ND) group (3.85 Kcal/g, 10% energy from fat, purified water), HFD group (5.24 Kcal/g, 60% energy from fat, purified water), HFD + OT group (5.24 Kcal/g, 60% energy from fat, OT). Both ND (D12450J) and HFD (D12492) groups were obtained from Xietong Co., Ltd. (Jiangsu, China). All drinking fluids were given to mice in sterile plastic bottles, which were replaced with fresh water or freshly prepared OT every 2 days, respectively, and the surplus was collected and measured. Individual body weights were recorded weekly, and the diet intake was also monitored every second day throughout the study. After 8-week treatment, all mice were euthanized to collect the serum, liver, adipose tissue, colon, and colonic content. All animal experiments were approved by the institutional animal care and use committee of Nankai University.

### Serum biochemical assessment

The levels of total cholesterol (TC), triglyceride (TG), low-density lipoprotein cholesterol (LDL-C), high-density lipoprotein cholesterol (HDL-C), alanine aminotransferase (ALT), aspartate aminotransferase (AST), lipopolysaccharide (LPS), interleukin-6 (IL-6), and tumor necrosis factor-alpha (TNF-α) in the serum were evaluated by using the corresponding kits. The oral glucose tolerance test (OGTT) in mice was carried out at the end of the eighth week after administration of glucose (2 g/kg.bw). The blood glucose levels in tail blood samples were evaluated at fasting and 2 h after the OGTT, and the area under the curve (AUC) was also calculated.

### Histological examination

The liver, epididymal adipose, and intestinal tissues were obtained and fixed overnight at 4°C in formalin solution. Randomly selected paraffin-embedded tissue slices from ND, HFD, and HFD + OT groups were stained with hematoxylin and eosin (H&E) and then viewed under a light microscope.

### RNA extraction and real-time quantitative polymerase chain reaction

The mRNA of inflammation and lipid metabolism-related genes extracted from the liver and adipose tissue was used as a template for reverse transcription with subsequent real-time quantitative polymerase chain reaction (RT-qPCR) according to Feng et al. with minor revisions (18). The specific primer sequences are shown in Table 1. Relative mRNA expression was calculated by using the 2^–ΔΔ*Ct*^ method and normalized to β*-actin* mRNA levels.

**TABLE 1:** Primer sequences for qPCR.

Gene	Forward primer (5′⟶3′)	Reverse primer (5′⟶3′)
*Ppar*α	TGCAGCCTCAGCCAAGTTGAA	TCCCGAACTTGACCAGCCA
*Hsl*	GCTAGCCAGGCTCATCTCCT	GTTCTTGAGGTAGGGCTCGT
*Fas*	GCTGCGGAAACTTCAGGAAAT	AGAGACGTGTCACTCCTGGACTT
*Hmgr*	TGCCTGGATGGGAAGGAGTA	GCACCTCCACCAAGGCTTAT
*Leptin*	CCTGTGGCTTTGGTCCTATCTG	AGGCAAGCTGGTGAGGATCTG
*Atgl*	ACAGCTCCAACATCCAC	AGCCCTGTTTGCACATCTCT
*Acox*	CTATGGGATCAGCCAGAAAGG	AGTCAAAGGCATCCACCAAAG
*Lxr*α	TCAGAAGAACAGATCCGCTTG	CGCCTGTTACACTGTTGCT
*Ppar*γ*1*	CCAGCATTTCTGCTCCACAC	ATTCTTGGAGCTTCAGGCCA
*Adiponectin*	CCCTGGTCTCCACGACTCTT	GCGAATATTGTGAAGCCCCC
*Acc*	GGCAGCAGTTACACCACATAC	TCATTACCTCAATCTCAGCATAGC
*Srebf1*	CTGGTGAGTGGAGGGACCAT	GACCGGTAGCGCTTCTCAAT
*Pgc1*α	AGCCGTGACCACTGACAACGAG	GCTGCATGGTTCTGAGTGCTAAG
*Tnf*-α	AATGGCCTCCCTCTCATCAG	CCACTTGGTGGTTTGCTACG
*Il-6*	ACTTCCATCCAGTTGCCTTCTTG	TGTTGGGAGTGGTATCCTCTGTG
*Il-1*β	AAGGGCTG TTCCAAACCTTTGAC	TGCCTGAAGCT TTGTTGATGTGC
β*-actin*	ACAGCAGTTGGTTGGAGCAA	ACGCGACCATCCTCCTCTTA

*Ppar*α, peroxisome proliferator-activated receptor alpha; *Hsl*, hormone-sensitive lipase; *Fas*, fatty acid synthase; *Hmgr*, 3-hydroxy-3-methyl-glutaryl-coenzyme A reductase; *Atgl*, adipose triglyceride lipase; *Acox*, acyl CoA oxidase; *Lxr*α, liver X receptors α; *Ppar*γ1, peroxisome proliferator-activated receptor γ1; *Acc*, acetyl-CoA carboxylase; *Srebf1*, sterol regulatory element-binding transcription factor 1; *Pgc1*α, peroxisome proliferator-activated receptor c coactivator 1α; *Tnf*-α, tumor necrosis factor α; *Il-6*, interleukin-6; *Il-1*β, interleukin-1β.

### Gut microbiota analysis

Genomic DNA of colonic contents was extracted, purified, and qualified, and then the V3–V4 hypervariable region of the bacterial 16S rRNA gene was amplified using universal primers (341F and 806R), as described previously (19). The alpha diversity and beta diversity were determined by Qiime (version 1.9.1) and principal coordinates analysis (PCoA), respectively. Spearman correlation analysis was conducted to explore the correlation between bacterial flora and physiological factors. Redundancy analysis/canonical correlation analysis (RDA/CCA) was also performed to analyze the relationship between gut microbial communities and obesity-related properties in R software (v. 2.15.3).

### Statistical analysis

Data were expressed as means ± standard errors of the means (SEM). The difference between two groups was analyzed by Student’s *t*-test when comparing the results at variable time points. The difference between the three groups was analyzed using one-way analysis of variance (ANOVA), and then the Tukey test was conducted for the comparison between groups using SPSS 23.0 and GraphPad Prism 5, and *p* < 0.05 was considered significant.

## Results

### Identification analysis of oolong tea

The main components of OT can be found in Table 2. Compared with tea protein (499.95 ± 52.79 μg/mL) and theanine (9.68 ± 6.63 μg/mL), OT contains markedly higher amounts of polyphenols (636.17 ± 8.54 μg GAE/mL). HPLC was applied to further determine the content of polyphenols including catechins and GA, the highest of which appears in the case of EC (700.78 ± 0.78 μg/mL), followed by GCG (21.06 ± 0.49 μg/mL). The results also demonstrated that the amounts of GC, EGCG, CG, ECG, C, and GA contained in the OT were 17.64 ± 0.42, 8.35 ± 0.32, 6.58 ± 0.22, 5.58 ± 0.35, 2.18 ± 0.27, and 31.35 ± 0.89 μg/mL, respectively. As mentioned in HPLC analysis, OT, which contains a relatively large amount of polyphenol, is thus considered a significant dietary source of health-promoting components.

**TABLE 2:** Main compositions of oolong tea.

Ingredients	Content
Protein	499.95 ± 52.79 μg/mL
Theanine	9.68 ± 6.63 μg/mL
Total phenolic content	636.17 ± 8.54 μg GAE/mL
Polyphenols	μg/mL
Catechins	762.17 ± 2.02
EC	700.78 ± 0.78
GCG	21.06 ± 0.49
GC	17.64 ± 0.42
EGCG	8.35 ± 0.32
CG	6.58 ± 0.22
ECG	5.58 ± 0.35
C	2.18 ± 0.27
Gallic acid	31.35 ± 0.89

EC, epicatechin; GCG, gallocatechin gallate; GC, gallocatechin; EGCG, epigallocatechin gallate; CG, catechin gallate; ECG, epicatechin gallate; C, catechin.

### Oolong tea reduced overweight and dyslipidemia in high-fat diet-fed mice

**FIGURE 1:**
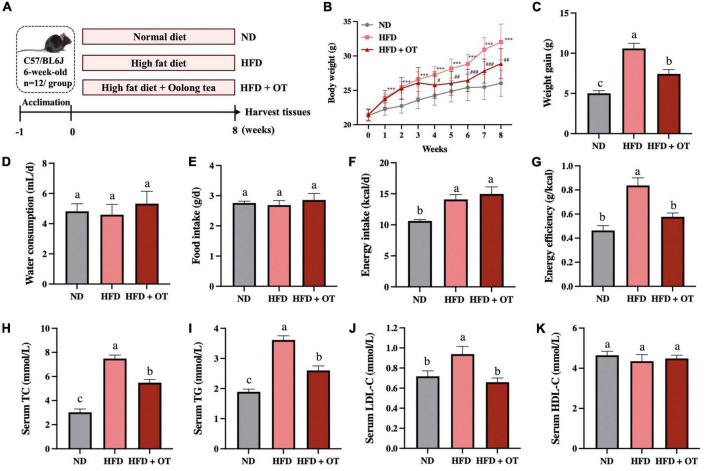
Oolong tea reduced overweight and dyslipidemia in HFD-fed mice. **(A)** Schematic overview of the animal experiment. **(B)** Body weight throughout the 8-week intervention. **(C)** Body weight gain. **(D)** Average daily water consumption. **(E)** Average daily food intake. **(F)** Energy intake. **(G)** Energy efficiency. **(H)** Serum TC levels. **(I)** Serum TG levels. **(J)** Serum LDL-C levels. **(K)** Serum HDL-C levels. Data are expressed as mean ± SEM (*n* = 12). **(A,C–K)** The mean value with different letters indicates significant differences (*p* < 0.05). **(B)** HFD vs. ND: ^∗∗∗^*p* < 0.001; OT vs. HFD: ^#^*p* < 0.05, ^##^*p* < 0.01, ^###^*p* < 0.001.

To better understand the functional role of OT in HFD-induced obesity, C57BL/6 mice were supplemented with OT under continuous HFD feeding for 8 weeks (Figure 1A). After 2 weeks, a significant increment in body weight was noticed in the HFD-fed mice compared with the control group, and this sharp rise persisted until the end of the intervention (Figure 1B), while OT supplementation inhibited HFD-induced overweight from week 4 onward (*p* < 0.05) and notably lowered the total weight gain in HFD-fed mice (Figure 1C). No evident change in water consumption and food intake was observed between different groups in Figures 1D,E, while decreased energy efficiency (weight gain/energy intake ratio) was seen in the HFD + OT group, indicating that energy efficiency was the primary driver of the anti-obesity effect of OT, rather than energy intake (Figures 1F,G).

Long-term HFD also induced serum dyslipidemia, increasing serum TC, TG, and LDL-C levels by 2.47, 1.90, and 3.46 times, respectively, while these HFD-induced higher levels of serum lipid were suppressed by OT supplementation (Figures 1H–J). Compared with normal diet-fed mice, no significant difference was found in HDL-C affected by HFD and OT feeding in mice (Figure 1K). These results suggested that OT intake effectively prevented HFD-induced overweight and dyslipidemia.

### Oolong tea alleviated lipid metabolism disorder in high-fat diet-fed mice

**FIGURE 2:**
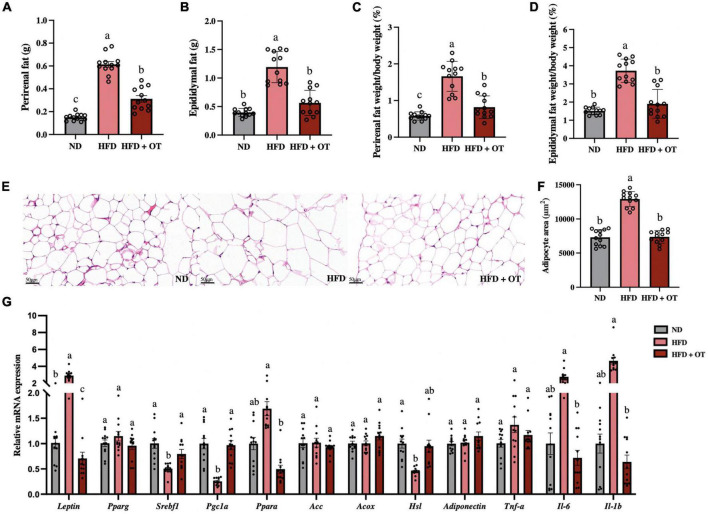
Oolong tea alleviated HFD-induced hypertrophy of white adipose tissue and lipid metabolism disorder. **(A)** Perirenal fat weight. **(B)** Epididymal fat weight. **(C)** Perirenal fat/body weight ratio. **(D)** Epididymal fat/body weight ratio. **(E)** Representative images of histological sections of epididymis adipose (scale bar, 50 μm). **(F)** Adipocyte area. **(G)** Relative expression of *Leptin*, *Pparg1*, *Srebp1c*, *Pgc1a*, *Ppara*, *Acc*, *Acox*, *Hsl*, *Adiponectin*, *Tnf*-α, *Il-6*, and *Il-1b* in the epididymal adipose tissue. Data are expressed as mean ± SEM (*n* = 12). The mean value with different letters indicates significant differences (*p* < 0.05).

As shown in Figures 2A–D, when comparing obesity-related traits, we found that HFD increased the weight of perirenal fat and epididymal fat, as well as their corresponding weight/body weight ratios, whereas these changes were substantially mitigated by OT intervention. Representative histology slices of epididymal adipose tissue and adipocytes area are also presented in Figures 2E,F, suggesting that OT significantly lowered the mean adipocyte size in mice on HFD.

To further understand the effects of OT intervention on lipid metabolism, we analyzed the expression of relevant genes in epididymal adipose tissue (Figure 2G). The expression of *Leptin* was significantly upregulated, while the expression of lipogenesis and lipid catabolism-associated genes including sterol regulatory element-binding transcription factor 1 (*Srebf1*), peroxisome proliferator-activated receptor gamma coactivator 1 alpha (*Pgc1a*), and hormone-sensitive lipase (*Hsl*) was significantly downregulated in the HFD group, as compared with the baseline level (*p* < 0.05). However, OT treatment significantly reversed the mRNA expressions of *Srebf1* and *Pgc1a* and suppressed the mRNA expression of *Leptin*, peroxisome proliferator-activated receptor alph*a* (*Ppara*), interleukin-6 (*Il-6*), and interleukin-1 beta (*Il-1b*). These findings indicated that OT intervention could influence lipid metabolism by regulating fat accumulation, lipid droplet aggregation, and abnormal expression of obesity-related genes.

### Oolong tea ameliorated fatty liver and impaired hepatic function in high-fat diet-fed mice

**FIGURE 3:**
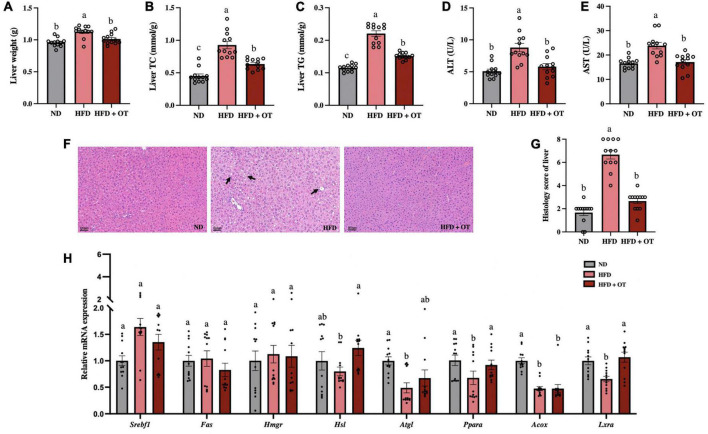
Oolong tea ameliorated fatty liver and impaired hepatic function in HFD-fed mice. **(A)** Liver weight. **(B)** Liver TC. **(C)** Liver TG. **(D)** Serum ALT levels. **(E)** Serum AST levels. **(F)** H&E staining of liver tissue (scale bar, 100 μm). **(G)** Histology score. **(H)** Relative expression of *Srebp1c*, *Fas*, *Hmgr*, *Hsl*, *Atgl*, *Ppara*, *Acox*, and *Lxra* in the liver. Data are expressed as mean ± SEM (*n* = 12). The mean value with different letters indicates significant differences (*p* < 0.05).

As observed in Figure 3A, the liver weight was significantly increased in HFD-fed mice compared with that in the ND group, while OT intervention remarkably lowered liver weight in mice on HFD (*p* < 0.05). OT also markedly reduced the levels of hepatic TC and TG in the HFD-fed mice (Figures 3B,C). Generally, serum ALT and AST levels are recognized as markers of liver function (20). Figures 3D,E showed that the elevated levels of ALT and AST in the HFD group were significantly ameliorated by OT supplementation, indicating its preventive effect on hepatotoxicity. H&E-staining-based histological analysis of liver sections was consistent with the biochemical parameters (Figure 3F). OT intervention improved HFD-induced indistinct cell boundaries, denaturation, and irregular damaged cells with cytoplasmic vacuolation (black arrows). The non-alcoholic fatty liver discase active score (NAS) can be found in Figure 3G, where the results demonstrated that HFD-fed mice developed hepatocyte ballooning, steatosis, lobular inflammation, and fibrosis, while the HFD + OT group showed normal histology, including the nucleus, intact cytoplasm, clear cell borders, and visible nucleolus.

Furthermore, to examine the changes in hepatic lipid metabolism regulated by HFD, the expression of lipid metabolism-related genes was determined (Figure 3H). The expression of genes including *Ppara*, adipose triglyceride lipase (*Atgl*), acyl CoA oxidase (*Acox*), and liver X receptors alpha (*Lxra*) was detected at lower levels in HFD-fed mice than in the ND group. However, OT supplementation significantly upregulated the mRNA expression of *Hsl*, *Ppara*, and *Lxra* in comparison to the HFD group (*p* < 0.05). These results suggested OT intervention can effectively attenuate HFD-induced liver steatosis, impair hepatic function, and differentially express liver metabolic genes.

### Oolong tea improved glucose tolerance and inflammatory disorder in obese mice

**FIGURE 4:**
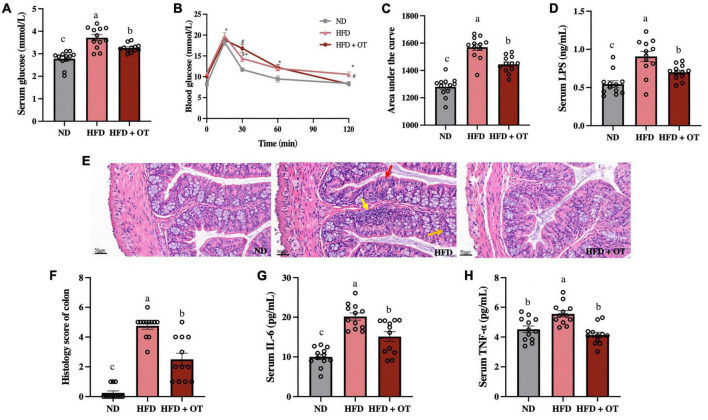
Oolong tea improved glucose tolerance and inflammatory disorder in obese mice. **(A)** Serum glucose levels. **(B)** Oral glucose tolerance test. **(C)** Calculated AUC for OGTT. **(D)** Serum LPS levels. **(E)** H&E staining sections from colon tissue (scale bar, 50 μm). **(F)** Histology score. **(G)** Serum IL-6 levels. **(H)** Serum TNF-α levels. Data are expressed as mean ± SEM (*n* = 12). **(A,C,D,F–H)** The mean value with different letters indicates significant differences (*p* < 0.05). **(B)** HFD vs. ND: ^∗^*p* < 0.05, ^∗∗^*p* < 0.01; OT vs. HFD: ^#^*p* < 0.05.

Figure 4A reveals that HFD feeding induced a marked increment in fasting blood glucose levels compared with those fed a normal diet (*p* < 0.01), whereas changes were substantially mitigated by OT intervention (*p* < 0.05). Moreover, the OGTT was conducted, and Figures 4B,C also show that compared to mice on normal diet, the HFD-fed mice exhibited higher blood glucose levels, which could be ameliorated by OT intervention after glucose injection, and the AUC of glucose in mice with OT feeding was significantly larger than that of HFD-fed mice (*p* < 0.05).

Furthermore, serum LPS levels were determined due to the emerging metabolomics evidence supporting the association between LPS and metabolic disorders (21). The results are given in Figure 4D, suggesting OT could inhibit HFD-induced significant increment in serum LPS levels (*p* < 0.05). It has also been reported that serum LPS levels could influence the anti-obesity effect of tea polyphenols by modulating intestinal inflammation (1). Subsequently, histopathological injury in the colon was measured to evaluate the effect of OT on colon pathological features and intestinal inflammation-related obesity (Figure 4E). HFD could lead to inflammatory infiltration including aggregated lymphocytes (yellow arrow), absence of neutrophils and wheel-like plasma cells in the crypt lumen (red arrow), and abnormal epithelium characterized by lower levels of goblet cells and mucin depletion (orange arrow), while OT could strikingly suppress these changes. According to the histology score (Figure 4F), the ND group appears to have normal colon histomorphology, but the results of HFD-fed mice exhibited inflammatory infiltrates and transmural injuries, while OT efficiently reduced the degree of intestinal mucosal damage.

In addition, Figures 4G,H show that OT could significantly prevent HFD-caused increased levels of TNF-α and IL-6 (HFD vs. ND, *p* < 0.001, *p* < 0.05; HFD + OT vs. HFD, *p* < 0.05, *p* < 0.05, respectively). Altogether, these results indicated that OT intervention could improve HFD-driven inflammation, glucose tolerance, and metabolic endotoxemia.

### Oolong tea supplementation attenuated high-fat diet-induced gut flora dysbiosis

**FIGURE 5:**
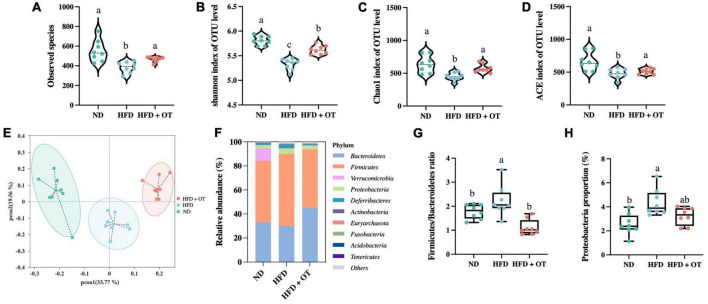
Oolong tea reshaped the gut microbiota components in HFD-fed mice. **(A)** Observed species. **(B)** Shannon index. **(C)** Chao1 index. **(D)** ACE index. **(E)** Weighted UniFrac PCoA. **(F)** Relative abundance of gut microbiota at the phylum level. **(G)** Firmicutes/Bacteroidetes ratio. **(H)** Relative abundance of Proteobacteria. Data are expressed as mean ± SEM (*n* = 8, randomly selected within the group). The mean value with different letters indicates significant differences (*p* < 0.05).

Considering the critical role of intestinal flora in the obesity-related diseases, we determined the gut microbial profile by 16S rRNA sequencing to investigate the preventive effect and underlying mechanisms of OT supplement on HFD-induced obesity. As shown in Figures 5A–D, HFD-fed mice showed lower levels of microbial diversity (Shannon and ACE index) and richness (observed species and Chao1 index) compared with ND group, while OT treatment significantly increased the levels of observed species, Shannon, ACE, and Chao 1 index in comparison to the HFD group. PCoA based on the operational taxonomic units (OTUs) level was also performed to investigate the β-diversity of the intestinal flora in different groups (Figure 5E). The separation of the HFD-fed mice and ND group showed the difference in microbial community composition, whereas OT reversed the gut dysbiosis observed in HFD-fed mice and altered the balance to one like that of the ND group.

To further explore the differences in the gut flora composition, the relative abundance of bacterial communities at the phylum and genus levels was analyzed. As shown in Figure 5F, at the phylum level, Firmicutes and Bacteroidetes were the predominant phyla, accounting for 51.22 and 33.06% of the intestinal flora in the ND group, and these two types of bacteria constituted 60.16 and 30.05% of the microbial profile in HFD-fed mice but changed to 48.65 and 45.20% under OT intervention. Figure 5G also demonstrates that OT could reverse and normalize HFD-caused imbalance of the Firmicutes/Bacteroidetes ratio (*p* < 0.01). Meanwhile, HFD-driven increment in the relative expression of Proteobacteria was significantly restrained by OT treatment (Figure 5H).

**FIGURE 6:**
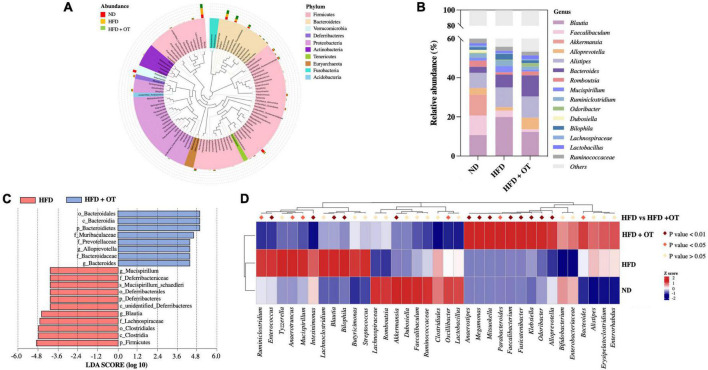
Oolong tea modulated microbial taxa in HFD-fed mice. **(A)** Species evolution tree at the phylum level. **(B)** Relative abundance of gut microbiota at the genus level. **(C)** Analysis of differences in the microbial taxa shown by LDA score (log10 > 4). **(D)** Heatmap showing the relative abundance of 35 OTUs in different groups (*n* = 8).

The differential expressed 10 phyla between HFD-fed mice and HFD + OT groups are identified in Figure 6A, and the differences in the community composition observed at the genus level among the investigated groups are represented in Figure 6B. Compared with ND-fed mice, OT beverage intake altered the relative abundance of microbial genera, including *Blautia*, *Alloprevotella*, *Mucispirillum*, *Odoribacter*, *Ruminiclostridium*, and *Bilophila*, and these significant changes were affected in the HFD + OT group. OT intervention also partially inhibited or completely reversed HFD-induced relative abundance reduction of some beneficial genera, including *Alloprevotella*, *Alistipes*, and *Lactobacillus*. Moreover, differentially abundant fecal bacterial taxa in HFD-treated mice in response to OT supplement were identified by linear discriminant analysis (LDA), represented in Figure 6C. *Muribaculaceae*, *Prevotellaceae*, *Bacteroidaceae*, *Bacteroides*, and *Alloprevotella* were significantly enriched in HFD-fed mice, and *Deferribacteraceae*, *Lachnospiraceae*, *Blautia*, and *Mucispirillum* were dominant in the OT-treated group. Heatmap in Figure 6D shows the relative abundance of top 35 OTUs in responding to the treatment of HFD and OT. Among 35 dominant OTUs, 19 OTUs were screened for significant changes due to OT intervention in HFD-fed mice (*p* < 0.05). In total, seven OTUs were abundant in the HFD group, while reversed by OT treatment to the baseline level (*p* < 0.05); four of those were selected to be the key phylotypes with the abundances altered significantly by OT treatment (*p* < 0.01) including *Enterococcus*, *Intestinimonas*, *Blautia*, and *Bilophila*, indicating OT could restore the HFD-disrupted intestinal flora composition toward those in the ND group.

### Correlation between gut microbiota and metabolic parameters

**FIGURE 7:**
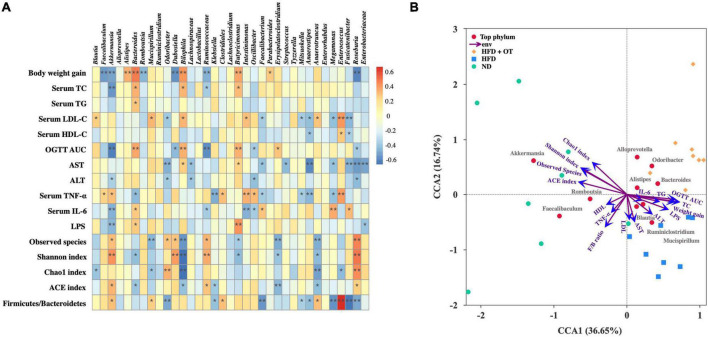
Correlation between gut microbiota and metabolic parameters. **(A)** Spearman’s correlation heatmap between gut microbiota and metabolic disorders-related indices. *p < 0.05, **p < 0.01. **(B)** CCA/RDA analysis (*n* = 8).

To identify the specific bacterial OTUs involved in the OT-mediated prevention on obesity, the correlation between the relative abundance (from the 35 most abundant OTUs) of the gut microbiota using Spearman correlation analysis was conducted. Figure 7A indicates 28 OTUs were associated with at least one obesity-related index. *Bacteroides*, *Bilophila*, and *Butyricimonas* positively correlated to weight gain, serum TC and IL-6 levels, and OGTT AUC; however, conversely, *Akkermansia* has a significantly negative correlation with weight gain, serum TC, ALT, LPS, IL-6 levels, and OGTT AUC (*p* < 0.05), suggesting that *Akkermansia* played an important role in OT attenuation on HFD-induced obesity, which was consistent with previous research (22). *Romboutsia*, *Lachnospiraceae*, *Roseburia*, *Odoribacter*, *Enterobacteriaceae*, *Anaerostipes*, *Megamonas*, *Mitsuokella*, and *Fusicatenibacter* were also negatively correlated with some obesity parameters. Meanwhile, these gut bacteria negatively associated with obesity phenotypes might be the effective bacterial genera contributing to the prevention of OT on obesity.

Based on the microbiome and obesity-related profile data, CCA/RDA analysis was applied to understand a possible correlation between different taxa and environmental factors during OT treatment in HFD-fed mice. It has been reported that the length of arrow represents the importance of environmental factors, while the angle between these indicators and samples indicates the correlation in the CCA/RDA analysis (23). CCA 1 and 2 accounted for 36.65 and 16.74% of the correlation between microbiota composition and obesity-related parameters (Figure 7B). The result also revealed that the indicators involving blood lipid, liver function, and microbial diversity found in HFD-fed mice significantly differ from those found in the ND group, while inflammatory indices presented less correlation with different taxa. These results indicated that the gut microbiota diversity and composition highly correlate with obesity-associated changes.

## Discussion

As the results of HPLC-MS/MS showed, the main catechins in OT included EC, GCG, GC, EGCG, and CG; especially, the content of EC reached 700 μg/ml, which concurs with previous reports (24). Studies have shown that the degree of fermentation has a significant impact on chemical components and tea quality (25). Thus, the moderate semi-fermentation of OT confers health-promoting antioxidant properties as well as excellent flavor and taste because the higher levels of tea polyphenols present in OT can effectively scavenge free radicals through phenolic hydroxyl group (26). In animal experiments, there was a significant anti-obesity effect observed in the HFD + OT group with no statistical difference in water consumption among groups, indicating good palatability and prebiotic function of OT in mice. Recently, the growing prevalence of obesity worldwide has become a significant public health concern. Although numerous studies have demonstrated that functional beverages rich in flavonoids and saponins can prevent obesity, which suggests the effectiveness of dietary intervention including bioactive ingredients enriched products, the regulatory effects and underlying mechanisms of OT, a good source of polyphenols, on obesity still need further investigations (27, 28).

Obesity is always accompanied by glucose–lipid metabolism disturbance and abnormal energy metabolism, regarded as early symptoms of diabetes (29). Our results suggested OT supplementation could remarkably attenuate HFD-induced obesity-related traits including higher levels of adipose weight, blood glucose, and serum lipid profiles; damaged histological appearance; and glucose tolerance, which is consistent with the anti-obesity effects of other tea species (green tea and black tea) (30, 31). Moreover, impaired hepatic homeostasis and low-grade systemic inflammation were also closely associated with the development of obesity. OT intervention was proved to prevent the toxicity as aforementioned by alleviating inflammatory infiltrates in both liver and colon and inhibiting excessive release of inflammatory factors, ALT, and AST.

The liver and adipose tissues are the main sites for lipid metabolism, where enzymes and adipokines regulating obesity, lipogenesis, and energy metabolism are released. Among them, *Ppars* including *Ppara*, *Pparb*, and *Pparg*, are a family of nuclear hormone receptors which play a critical role in regulating lipid and fatty acid metabolism (32, 33). *Ppara* has been found to affect β-oxidation of fatty acid accompanied by the stimulation of *Pgc1a* from adipose tissue (34). In addition, lipid homeostasis is precisely regulated by diverse molecules, such as *Srebf1*, that promotes *de novo* lipogenesis (DNL) and fatty acid intake (35). It is also well-documented that *Srebf1* exhibits an essential role in the activation of DNL-related enzymes including *Acc*, *Fas*, and *Lxra* regarded as a regulatory sensor for lipid metabolism (36). In addition to energy storage, adipose tissue functions as an endocrine organ releasing various adipokines (e.g., leptin and adiponectin) with a wide range of physiological effects on obesity-related symptoms (37). The prolonged exposure to HFD could lead to hyperleptinemia accompanied by central leptin resistance, resulting in unbalanced energy homeostasis (38). OT intervention repressed leptin expression, which was consistent with a population-based study where leptin levels were negatively correlated with obesity traits (39). Otherwise, two types of lipolytic enzymes, *Hsl* and *Atgl*, are involved in the appetite regulation and lipid metabolism (40). Chronic low-grade inflammation characterized by higher levels of *Il-1b* and *Il-6* has been reported to be one of the hallmarks of obesity-induced insulin resistance (41). Overall, our findings showed HFD might influence the process of β-oxidation of fatty acid (*Ppara*, *Acox*, and *Pgc1a*), lipolysis (*Atgl*, *Srebf1*, and *Hsl*), cholesterol removal (*Lxra*), and appetite regulation (*Leptin*), leading to lipid metabolism disorder. However, OT intervention exerted a preventive effect on obesity possibly by regulating β-oxidation of fatty acid (*Ppara* and *Pgc1a*), cholesterol removal (*Lxra*), lipolysis (*Srebf1* and *Hsl*), and low-grade inflammation (*Il-6* and *Il-1b*).

Regulation of the microbiome profile has been identified as a critical strategy in the prevention/treatment of obesity. Our findings suggested that OT recovered an HFD-led reduction in the gut flora richness and diversity, coupled with an increase in the Firmicutes/Bacteroidetes ratio and the relative abundance of Proteobacteria, indicating the protective effect of OT on gut flora disorder (42). By comparing the intestinal flora composition at the genus level, we found that OT could reverse the differential abundance of *Blautia*, *Mucispirillum*, *Odoribacter*, *Ruminiclostridium*, and *Bilophila* caused by HFD feeding. Zhao et al. reported that the combination of quercetin and resveratrol could prevent HFD-driven obesity by downregulating the abundance of *Bilophila* (43), whereas the positive correlation between obesity and relative abundance of *Odoribacter* and *Ruminiclostridium* was also confirmed (44). Recently, *Mucispirillum* has been considered to interfere with obesity by the involvement in the mitochondrial energy metabolism and inflammatory processes, while *Oscillibacter* is closely related to HFD-induced obesity and reduces the expression of the intestinal mucosal barrier-related protein (45, 46). In addition, OT could regulate the relative abundance of some beneficial genera including *Alloprevotella*, *Alistipes*, and *Lactobacillus*. Therefore, *Alloprevotella* and *Alistipes*, the producers of short-chain fatty acids (SCFAs), exert an anti-obesity effect by contributing to intestinal barrier integrity and increasing energy expenditure, while Lactobacillus was negatively correlated with obesity, dyslipidemia, and hypertension (47). In addition, seven differentially changed genera caused by HFD-feeding were reversed in the HFD + OT group, of which four strains showed significantly changed abundance including *Enterococcus*, *Intestinimonas*, *Blautia*, and *Bilophila*, indicating their significant roles in prevention/treatment of obesity. It has been reported that probiotic *Enterococcus* could mitigate obesity and produce propionic acid-stimulated apoptosis in 3T3-L1 pre-adipocytes (48). *Intestinimonas* was indicated to have relatively higher abundance in the inflammatory animal model, and *Blautia* was associated with fat accumulation and obesity in a clinical trial (49, 50). Also, *Bilophila* was proved to aggravate HFD-induced inflammatory and metabolic diseases in previous research (51). Moreover, those phylotypes enriched in the HFD + OT group including Odoribacter, Enterobacteriaceae, Anaerostipes, Megamonas, Mitsuokella, and Fusicatenibacter and negatively correlated with some obesity parameters and might be the effective bacterial genera contributing to the preventive effect of OT on obesity. Hence, alleviation of OT on HFD-induced obesity might be partially attributed to the modulation of the gut microbiota composition. Nevertheless, further investigation should be conducted to validate gut microbiota functionality.

As discussed before, OT as a good source of polyphenols, presented an anti-obesogenic effect in HFD-fed mice on the body weight gain, fat accumulation, glucose tolerance, serum lipid parameters, hepatic function, systematic inflammation, endotoxemia contents, mRNA expression levels of lipid metabolism-related genes, and gut flora disorder. Mechanically, we speculate OT is mainly metabolized and exerts anti-obesity effects in following two ways: (i) Role in internal organs: bioactive components could regulate the release of inflammatory factors (TNF-α and IL-6) coupled with the expression of catabolism genes including *Srebf1*, *Pgc1*α, *Hsl*, *Leptin*, and *Ppar*α in the liver and adipose tissue, thus reducing lipid accumulation, improving impaired hepatic homeostasis, and inhibiting systemic inflammation; (ii) Role in gastrointestinal tract: simple bioactive components such as GA are known to be absorbed in the small intestine, then diffuse through enterocytes, and reach hepatocytes, where they are converted into various metabolites used to regulate lipid metabolism and ameliorate inflammation (52). Complex bioactive components from OT including catechin, theanine, and tea protein may be digested into intermediate compounds and then interact with gut microbiota. Some intermediate products might be transported from the colon to the liver, where they are bio-transformed into glucuronide, sulfate, and methyl metabolites. Others unavailable for intestinal absorption might increase the diversity of microbial communities, positively alter intestinal flora composition, and modulate abundance of core microbes, thus resulting in a positive impact on host metabolic health and obesity-related diseases.

## Conclusion

In this study, OT intervention ameliorated HFD-induced weight gain, dyslipidemia, impaired hepatic homeostasis, fat accumulation, endotoxemia, and glucose intolerance, thus contributing to the prevention and treatment of obesity and gut microbial dysbiosis. Furthermore, the suppressed expression of inflammatory factors, regulatory expression of catabolism genes including *Srebf1*, *Pgc1a*, *Lxra*, *Hsl*, *Leptin*, and *Ppara*; the modulatory effect of microbial diversity; and richness were responsible for the preventive mechanisms against obesity of OT. OT also altered the HFD-induced differential abundance of the certain core microbes including *Enterococcus*, *Intestinimonas*, *Blautia*, and *Bilophila*. Some phylotypes enriched in the HFD + OT group, including *Odoribacter*, *Enterobacteriaceae*, *Anaerostipes*, *Megamonas*, *Mitsuokella*, and *Fusicatenibacter* and negatively correlated with some obesity parameters might be the effective bacterial genera contributing to the preventive effect of OT on obesity. This discovery may offer a new nutrition strategy to relieve obesity-related diseases, suggesting OT has great potential to be used as a functional beverage against obesity and metabolic disorders.

## Data availability statement

The original contributions presented in this study are included in the article/supplementary material, further inquiries can be directed to the corresponding author.

## Ethics statement

The animal study was reviewed and approved by the Institutional Animal Care and Use Committee of Nankai University and carried out in accordance with the National Institutes of Health guide for the care and use of laboratory animals (NIH Publications No. 8023).

## Author contributions

AL performed the experiments, analyzed the data, and prepared the manuscript. JW contributed to design of the study, built the animal model, and revised the manuscript. RK and MC were responsible for methodology. BZ, YZ, and JL coordinated the laboratory work. XX and BP modified the format. SW aided in the analysis and interpretation of the data. All authors agreed to be accountable for the content of the work and approved the manuscript.

## Abbreviations

HFD: high-fat diet
CVD: cardiovascular disease
FMT: fecal microbiota transplant
OT: oolong tea
HPLC: high-performance liquid chromatography
GA: gallic acid
EC: epicatechin
GCG: gallocatechin gallate
GC: gallocatechin
EGCG: epigallocatechin gallate
CG: catechin gallate
ECG: epicatechin gallate
C: catechin
ND: normal-diet
TC: total cholesterol
TG: triglyceride
LDL-C: low-density lipoprotein cholesterol
HDL-C: high-density lipoprotein cholesterol
ALT: alanine aminotransferase
AST: aspartate aminotransferase
LPS: lipopolysaccharide
IL-6: interleukin-6
TNF-α: tumor necrosis factor-alpha
OGTT: oral glucose tolerance test
AUC: area under the curve
H&E: hematoxylin and eosin
qPCR: quantitative real-time polymerase chain reaction
PCoA: principal coordinates analysis
RDA/CCA: redundancy analysis/canonical correlation analysis
OTUs: operational taxonomic units
LDA: linear discriminant analysis
DNL: *de novo* lipogenesis
SCFAs: short-chain fatty acids.
